# ERCC6L promotes the progression of hepatocellular carcinoma through activating PI3K/AKT and NF-κB signaling pathway

**DOI:** 10.1186/s12885-020-07367-2

**Published:** 2020-09-05

**Authors:** Han Chen, Hengxiao Wang, Xiqiao Yu, Shuping Zhou, Yueying Zhang, Zhaopeng Wang, Shuhong Huang, Zhaoxia Wang

**Affiliations:** Institute of Basic Medicine, Shandong First Medical University & Shandong Academy of Medical Sciences, Jinan, 250062 China

**Keywords:** ERCC6L, Hepatocellular carcinoma, PI3K, AKT, Oncogene

## Abstract

**Background:**

Excision Repair Cross-Complementation group 6-like (ERCC6L) has been shown to exhibit carcinogenic effect in several malignant tumors. However, the function and molecular mechanism of the ERCC6L in hepatocellular carcinoma (HCC) have not been investigated extensively.

**Methods:**

Immunohistochemistry analyses were used to detect ERCC6L expression in a HCC tissue microarray, and the Chi-square test was used to assess the correlation between ERCC6L expression and patients’ clinicopathological features. shRNA was used to down-regulation ERCC6L expression in HCC cell lines. MTT assay, plate clone formation assay, flow cytometry, caspase 3/7 activity and migration assays were performed to evaluate the impact of ERCC6L on HCC cells in vitro. Nude mice xenograft models were used to assess the role of ERCC6L in vivo. The regulatory of mechanism of PI3K/AKT pathway was evaluated by western blotting.

**Results:**

ERCC6L was highly expressed in HCC tissue compared with tumor adjacent tissues in 90 paired samples. ERCC6L expression positively correlated with gender, tumor encapsulation, and pathological stage. Patients with low ERCC6L expression had significantly longer OS than those with high ERCC6L expression. Knockdown of ERCC6L expression significantly inhibited proliferation, invasion and metastasis in vitro and tumor growth in vivo, and it promoted cell cycle arrest and apoptosis. Mechanistic analyses revealed that PI3K/AKT and NF-κB signaling pathway were inhibited by silencing ERCC6L.

**Conclusion:**

These results demonstrate that ERCC6L plays a critical role in HCC progression, and thereby might be a potential therapeutic target for HCC patients.

## Background

Hepatocellular carcinoma (HCC) is one of the most prevalent malignant tumors and the third most common cause of cancer-related deaths worldwide, often accompanied with invasive fast growing and metastasis [1, 2]. Although therapeutic strategies including surgery resection, liver transplantation and radiotherapy have greatly improved [3], there are limited effective therapies for patients with advanced HCC. The five-year post-surgical OS rate remains approximately 16% even after curative resection [4]. The main causes of poor clinical outcomes in patients with HCC are high recurrence and metastasis rates [5]. Therefore, the discovery of novel therapeutic target and better understanding of the molecular mechanisms underlying HCC progression are crucial and urgent.

Excision repair cross-complementation group 6-like (ERCC6L), also named as Polo-like kinase 1-interacting checkpoint “helicase” (PICH), which was identified as an embryonic development related proteins, has been shown to play critical roles in regulating the development of embryonic, brain and other tissues [6–8]. Recently several reports revealed that abnormal ERCC6L expression has been detected in several malignant solid tumors consisting of breast cancer [9], kidney cancer [10], and neuroblastoma [11]. In addition, high ERCC6L expression level is related to poor prognosis in breast cancer patients, and may regulate cell proliferation, invasion and metastasis by regulating different single pathways. However, the roles of ERCC6L in HCC progression and its underlying molecular mechanisms remain unclear.

In this study, we explored the effects of ERCC6L on the progression of HCC and confirmed ERCC6L to be a poor prognostic marker. In addition, we assessed the function of ERCC6L in tumor cell proliferation, apoptosis, migration, and molecular mechanisms in vitro and in vivo. These data proposed a potential target for prognosis and therapy in HCC patients.

## Methods

### Cell culture and transfection

Three human HCC cell line (SMMC7721, HuH-7, Hep3B) and a normal human liver cell line HL-7702 were obtained from the Institutes of Cell Biology at the Chinese Academy of Science (Shanghai, China). The cells were recently authenticated by STR profiling and are free from mycoplasma contamination. Cells were maintained in DMEM (Gibco, Grand Island, NY, USA) supplemented with 10% fetal bovine serum, 100 U/mL penicillin, and 100 mg/mL streptomycin (Gibco, Grand Island, NY, USA) in a humidified atmosphere with 5% CO_2_ at 37 °C. Transfection of shRNA (RiboBio Co., Ltd., Guangzhou, China) was performed with the transfection reagent in 2 nM shRNA according to the manuscripts of Lipofectamin 3000 (Invitrogen, Carlsbad, USA). Target sequences as following:

*ERCC6L* shRNA: 5′-GGACCATATTGATCAAGTA-3′;

Negative control shRNA (NC): 5′-TTCTCCGAACGTGTCACGT-3′.

The ERCC6L cDNA was cloned into a GV219 vector (GenePharma Co. Ltd., Shanghai, China) to overexpress ERCC6L. Cells were transfected for 48 and 72 h, then ERCC6L mRNA and protein expression were verified by quantitative real-time PCR or western blotting analysis respectively [12]..

### Total RNA extraction and quantitative real-time PCR

Total RNA was isolated from cells using TRIzol reagent (Invitrogen) [13]. 1 μg RNA was reversely transcribed into cDNA using Superscript First-strand Synthesis system (Invitrogen, Carlsbad, USA) according to the manufacturer’s instrucions. Quantitative RT-PCR was performed using SYBR Premix Ex Tag II (Takara Bio Inc.) on a Roche 4800 instrument (Applied Biosystems, USA). The primers were following:

ERCC6L*,* forward: 5′-AAGGATGAACGGACCAGAAAC-3′,

reverse: 5′-CTGTGAGGAGGAGGCGATTAC-3′;

β-actin, forward: 5′-AGAGCTACGAGCTGCCTGAC-3′,

reverse: 5′- AGCACTGTGTTGGCGTACA-3′.

The experiment was repeated three times, and all data were analyzed by the 2^-ΔΔCT^ method.

### Western blotting

Cell lysates were separated by 10% SDS-PAGE and transferred onto polyvinylidene difluoride (PVDF) membranes (Millipore). The membranes were blocked in 5% BSA for 1 h, then incubated with primary antibodies overnight at 4 °C. Antibodies were used as following: ERCC6L (Proteintech, 15,688–1-AP, 1:1000), PI3K (Bioss, bs-0128R, 1:1000), p-PI3K (Ser1070; Bioss, bs-6417R, 1:1000),AKT1 (Bioss, bs-0115R, 1:1000), p-AKT1 (Thr34; Bioss, bs-5194R, 1:1000), JAK2 (Bioss, bs-23003R, 1:1000), p-JAK2 (Tyr1007 + Tyr1008; Bioss, 2485R, 1:1000), NF-κB (Bioss, bs-0465R, 1:1000), p-NK-κB (Thr505; Bioss, bs-5663R, 1:1000), and β-actin (Bioss, bs-0061R, 1:5000). The next day membranes were incubated with HRP-conjugated secondary antibodies for 1 h at 37 °C. After washed for 3 times in TBST for 5 min, membranes were visualized by chemiluminescence kit and scanned with QuantityOne software (Bio-Rad, Hercules, CA, USA). The bands were analyzed with ImageJ (NIH, Bethesda, MA, USA).

### Migration assays

Migration assays were performed using a Transwell assay (8.0 μm, 24well, BD Biosciences). Briefly, 1 × 10^5^ cells were added into the upper chambers with serum-free DMEM, and the lower chambers were filled with 600 μL DMEM contained 10% FBS. After incubated for 24 h, the cells that migrated were fixed for 30 min and stained with 0.05% crystal violet. The numbers of migrating cells were counted in five randomly selected visual fields under a microscope at 200× magnification [14]. The experiments were performed three times.

### MTT assays

Cells were inoculated into a 96-well plate at 2 × 10^3^ cells/well and cultured for 24, 48, and 72 h respectively. Then, 20 μL of 5 mg/mL MTT solution (Sigma, USA) was added and incubated for 4 h. 150 μL of dimethyl sulfoxide (DMSO) was added and the optical density (OD) value was measured at 490 nm of the microplate reader. he experiments were performed three times separately.

### Cell colony formation

Cells were seeded into 6-well plates at a density of 500 cells/well for 14 days. Then cells were fixed with 4% paraformaldehyde for 30 min, and stained with 0.05% crystal violet for 20 min. Representative photographs were captured, and colonies with more than 40 cells were counted.

### Cell cycle analysis

For cell cycle analysis, cells transfected with shERCC6L or NC for 24 h. Cells were fixed in 70% ethanol, and stained with 50 μg/mL PI for 30 min in the dark at 37 °C. The percentage of cells in the G1, S, and G2 phases were determined with a FACS flow cytometer (Becton Dickinson, San Jose, USA).

### Apoptosis assay

Apoptosis was evaluated by flow cytometry using an Annexin V/FITC-PI Apoptosis Detection Kit (Millipore, USA) according to the manufacturer’s instructions. After washing with cold PBS, tumor cells were stained with 10 μL of Annexin V-FITC/PI in the dark for 15 min at room temperature and 400 μL of binding buffer was added. The cells were analyzed by flow cytometry (BD Biosciences, MA, USA). The Q2 and Q3 quadrants were determined as the early and late apoptosis induced cell, respectively. The percentage of total apoptosis (early and late stages) was used for quantitative analysis.

### Caspase-3/7 activity

Cell were transfected for with shERCC6L or NC 24 h, then collected and incubated with the working solution provided in the Caspase-Glo® 3/7 Assay Kit (Promega) according to manufacturer’s protocol [15].

### Immunohistochemistry (IHC)

The tissue microarray (HLivH180Su18, Outdo Biotech) was used to validate ERCC6L expression in 90 HCC tissue samples and tumor-adjacent tissues. Briefly, the samples were dewaxed, and antigens were retrieved using a microwave. The paraffin section were dewaxed with xylene (10 min × 3 times), followed by rehydration with gradient ethanol (anhydrous ethanol, 95% ethanol, 90% ethanol, 80% ethanol, 70% ethanol, 5 min each), and then antigens were retrieved using microwave for 15 min. After elimination of endogenous peroxidase activity, sections were blocked with 5% bovine serum albumin and then incubated with primary antibodies against ERCC6L (Bioss, bs-6380R, 1:100) overnight at 4 °C. Then sections were incubated with secondary antibodies (horseradish peroxidase (HRP)-conjugated goat anti-rabbit IgG (A0208, Beyotime, China) for 1 h. Sections were stained with Diaminobenzidine (DAB) solution and observed under a microscope (Leica DM4000B, Germany) as previously described [16].

Two pathologists who were separately blinded to the clinicopathological data evaluated the stained results independently. Staining intensity was scored as follows [17, 18]: 0, negative; 1, weak; 2, moderate; and 3, strong. The percentage of positive-stained cells was divided according to density: 0–10%; 11–25%; 26–50%; 51–75%; ≥ 76%, and score was 0, 1, 2, 3, 4 respectively. The total scores was calculated by multiplying the intensity scores by the percentage scores; total scores of 0–3 or 4–12 were defined as low or high expression.

### Xenografts tumor growth

BALB/c nude mice were obtained from Beijing Vital River Laboratory Animal Technology Co., Ltd. (male, 6 weeks old, 18-20 g). Animal experiments were approved by the Institute of Basic Medicine, Shandong First Medical University & Shandong Academy of Medical Sciences and were performed according to the established guidelines. Mice were fed under the condition of specific pathogen-free. shERCC6L and NC were used to construct lentiviral particles (GenePharma), and the lentivirus were infection to establish stable transfected cells. Mice were randomly grouped two groups (with 7 mice each group): negative control group (NC group, injected with SMMC7721 transfected with NC) and shERCC6L group (injected with SMMC7721 transfected with shERCC6L). A total of 1 × 10^7^ cells were suspended in 100 μL serum-free DMEM and injected subcutaneously into the right back of mice. After injection, the long diameter (L), width diameter (W) of tumors were measured every 5 days, and the volumes of tumor were calculated as following Volume (V) = (W^2^ × L)/2. Mice were anaesthetized by intraperioneal injection of pentobarbital sodium overdose (100 mg/kg) on the 30th day. Euthanasia was considered to be successful if there was cardiac arrest and no spontaneous breath for 3 min, and then tumors were taken out and weighed.

### Statistical analysis

All statistical analyses were performed using SPSS 22.0 Software (NY, USA). All measured data were presented as the mean ± SD, and Student’s *t*-tests were used for comparing the significance of differences between two groups. OS were calculated using the Kaplan–Meier method, and survival curve comparisons were performed using the log-rank test. Univariate and multivariate Cox regression was used for analysis factors predicting OS. *p* < 0.05 was considered the statistical significance.

## Results

### ERCC6L expression is upregulated in HCC patients

**Fig. 1:**
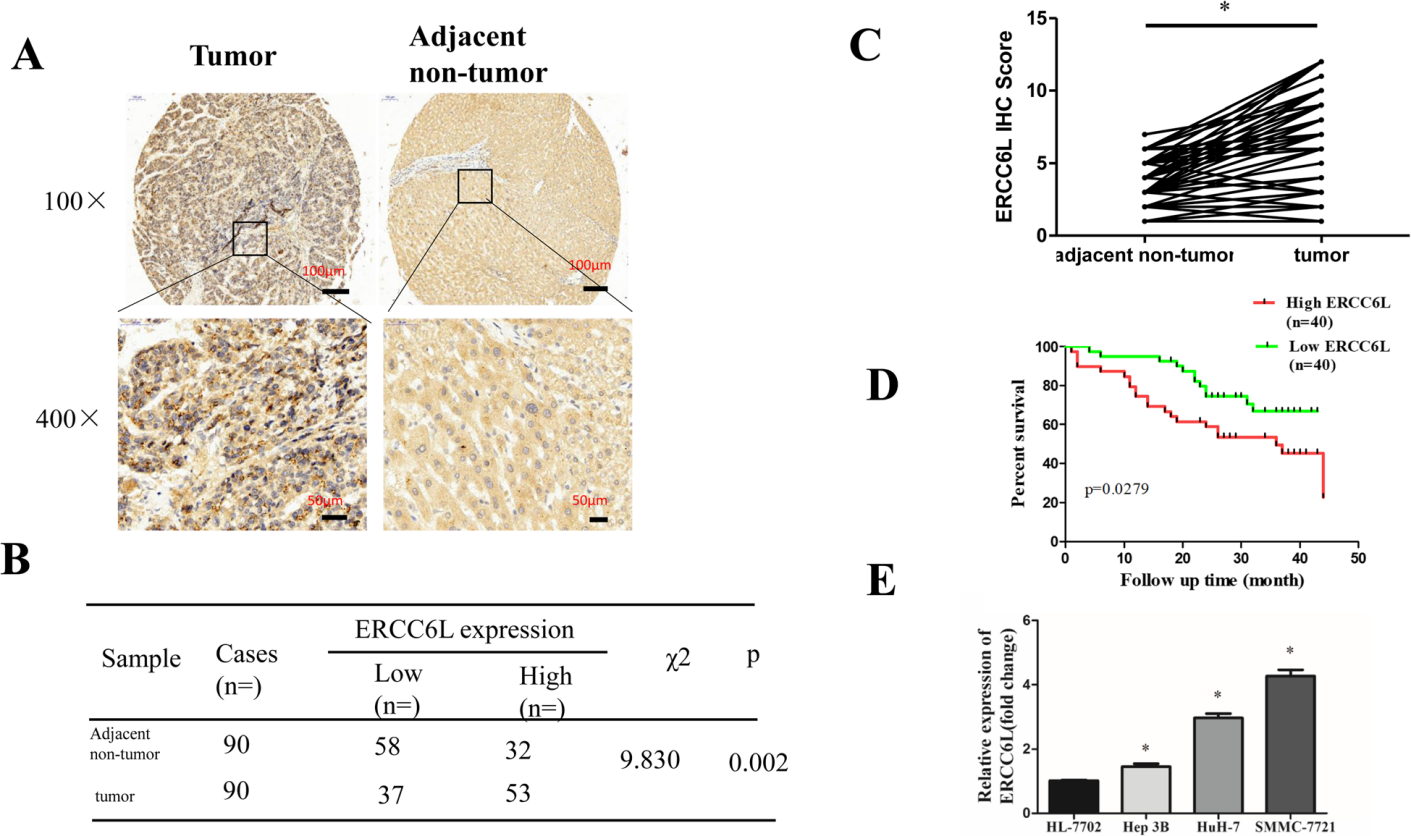
ERCC6L expression in upregulated in HCC. **a** Representative pictures of IHC staining on the tissue chip of HCC for ERCC6L. **b** Chi-square analysis of ERCC6L expression according to the IHC results in HCC tumor and adjacent non-tumor tissue. **c** Paired HCC tissue IHC scores for 90 patients (tumor versus adjacent non-tumor). **d** Overall survival of HCC patients with different levels of ERCC6L expression. **e**. ERCC6L mRNA expression in Hep3B, HuH-7, and SMMC-7721 cells compared to HL-7702 cells, *p < 0.05. All of the expression levels were normalized to β-actin levels. All experiments were carried out at least three independent times

To investigate the clinical significance of ERCC6L expression in patients with HCC, we examined its expression in a human HCC tissue array consisting of 90 paired HCC and tumor-adjacent tissues by IHC. As shown in Fig. 1a, ERCC6L was localized in the cytoplasm of HCC cells, and its expression was evidently higher in tumor tissue than in adjacent tissues (*p* < 0.05, Fig. 1b-c). We then divided the patients into high or low ERCC6L expression groups according to the IHC staining score and evaluated overall survival (OS) by Kaplan-Meier analysis. Compared with the low ERCC6L expression group, patients with high ERCC6L expression exhibited markedly shorter overall survival time (*p* < 0.05; Fig. 1d).

To investigate whether ERCC6L protein expression was associated with HCC progression, we analyzed the relationship between ERCC6L expression and clinical characteristics. ERCC6L expression positively correlated with gender, tumor encapsulation, and pathological stage (*p* = 0.012, 0.004, and 0.027, respectively), but not with age, liver cirrhosis, tumor size, tumor number (Table 1.). Univariate analysis showed that ERCC6L, tumor size and pathological stage were correlated with OS in HCC patients. In addition, a multivariate analysis demonstrated that the ERCC6L (HR 0.437, *p* = 0.045), the tumor size (HR 0.449, *p* = 0.037) and gender (HR 2.970, *p* = 0.026) were independent prognostic indicators for HCC patients (Table 2.).Taken together, these data suggest that ERCC6L expression levels are positively correlated with poor OS in HCC patients, indicating that ERCC6L are prognostic indicators for HCC.

**Table 1:** Correlation between ERCC6L protein expression in HCC and the clinical characteristics of HCC patients (*n* = 90)

Clinical characteristics	n	ERCC6L expression		*X*^*2*^	*P*
		Low	High		
Age, years					
< 50	27	9	16	0.207	0.810
≥ 50	63	26	37		
Gender					
Female	16	2	14	6.580	**0.012**
Male	74	35	39		
Liver cirrhosis					
No	73	32	41	0.475	0.584
Yes	17	5	12		
Tumor size, cm					
< 5	31	15	16	1.034	0.370
≥ 5	59	22	37		
Tumor number					
Single	83	32	51	2.882	0.119
Multiple	7	5	2		
Tumor encapsulation					
Complete	65	33	32	9.016	**0.004**
None	25	4	21		
Pathological stage					
I-II	64	31	33	4.912	**0.027**
III-IV	26	6	20

**Table 2:** Univariate and multivariate Cox regression analyses of overall survival

Clinical characteristics	Univariable analysis		Multivariabl analysis	
	HR (95%)	*P* value	HR (95%)	*P* value
Age, years (< 50 vs. ≥ 50)	0.820 (0.441–1.526)	0.532	1.143 (0.592–2.207)	0.691
Gender (Female vs. male)	2.253 (0.885–5.737)	0.089	2.970 (1.141–7.730)	0.026
Liver cirrhosis (No vs. Yes)	0.577 (0.244–1.368)	0.212	0.787 (0.311–1.991)	0.613
Tumor size, cm (< 5 vs. ≥ 5)	0.365 (0.176–0.758)	0.007	0.449 (0.211–0.953)	0.037
Tumor number (Single vs. Multiple)	1.436 (0.512–4.025)	0.492	2.242 (0.725–6.930)	0.161
Tumor encapsulation (Complete vs. None)	1.245 (0.662–2.341)	0.496	0.945 (0.481–1.860)	0.871
Pathological stage (I-II vs. III-IV)	2.134 (1.173–3.881)	0.013	1.729 (0.860–3.478)	0.124
ERCC6L (high vs. low)	0.463 (0.234–0.917)	0.027	0.437 (0.195–0.982)	0.045

### ERCC6L down-regulation suppresses tumor cell proliferation and migration in vitro

**Fig. 2:**
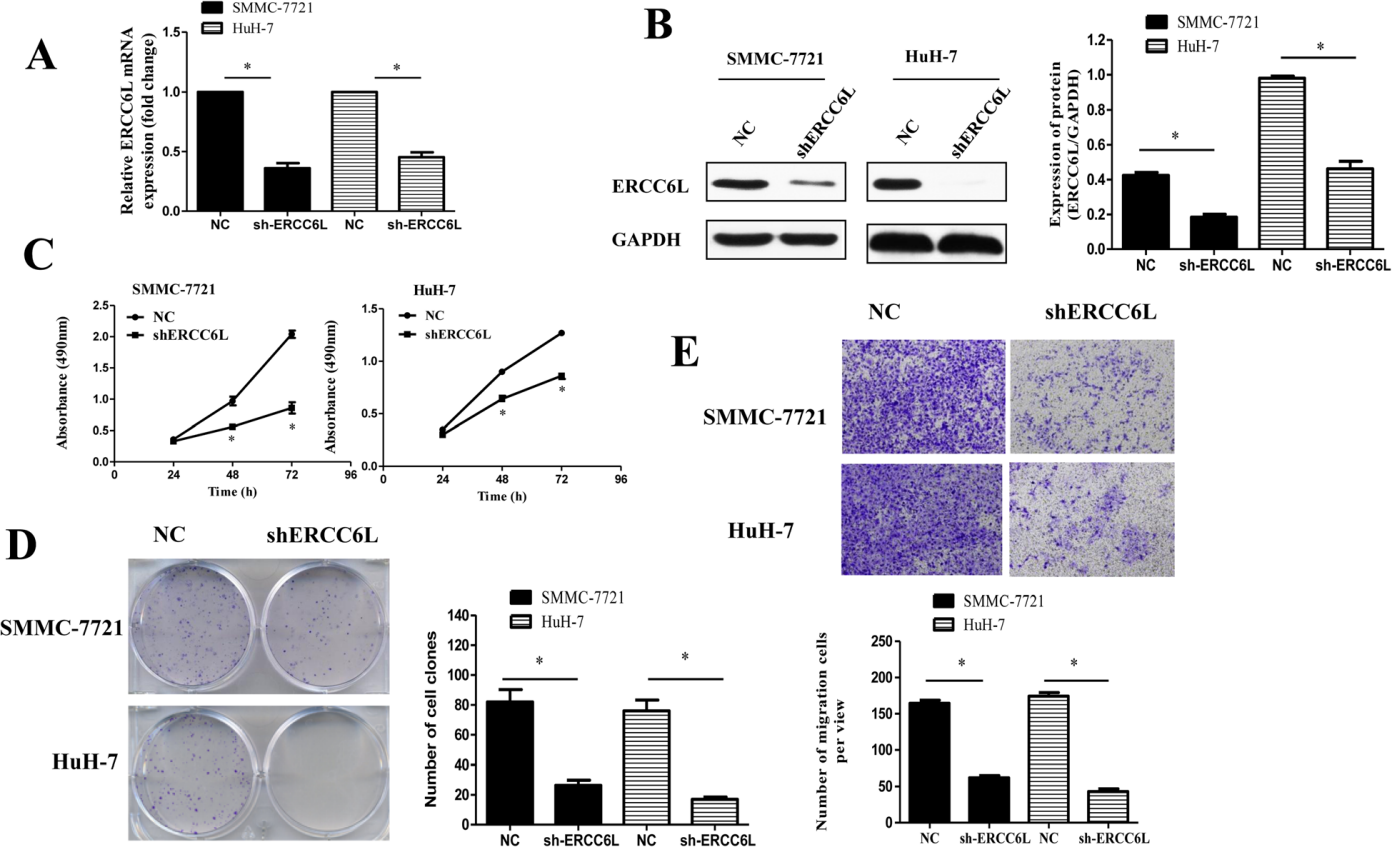
Effect of knockdown ERCC6L expressin on HCC cells proliferation and migration in vitro. **a**. ERCC6L protein expression in SMMC7721 and HuH-7 cells transfected with shERCC6L or NC, as determined by Western blotting, *p < 0.05. **b**. ERCC6L mRNA expression was determined by RT-PCR. *p < 0.05. **c**. Proliferation of SMMC7721 and HuH-7 cells transfected with shERCC6L or NC, as determined by MTT assays, *p < 0.05 compared with NC at the same time point. **d** The clone formation assay detected cell proliferation after transfecting with ERCC6L shRNA. **e**. Migration of SMMC7721 and HuH-7 cells transfected with shERCC6L or NC, as determined by Transwell assays, *p < 0.05 compared with the respective NC group. Dot plots represent one of three independent experiments. In the histogram, data are mean ± SD of three experiments

To explore the biological function of ERCC6L in HCC, we performed RT-qPCR to determine ERCC6L mRNA expression levels in three HCC cells lines and a normal liver cell line. As shown in Fig. 1e, ERCC6L mRNA level was significantly elevated in HCC cell lines than that of HL-7702 cell lines, particularly SMMC-7721 and HuH-7 cells (*p* < 0.05). Thus, SMMC-7721 and HuH-7 cells were used in subsequent experiments. A shR-ERCC6L-mediated loss-of-function approach was used to knockdown ERCC6L expression in SMMC7721 and HuH-7 cells. qRT-PCR and Western blotting results confirmed that the expression of ERCC6L mRNA and proteins were significantly decreased in SMMC7721 and HuH-7 cells transfected with shERCC6L than NC group (*p* < 0.05; Fig. 2 a-b).

MTT assays were used to measure cell proliferation. As shown in Fig. 2c, the proliferation of SMMC-7721 and HuH7 cells transfected with shERCC6L was significantly lower than that of NC cells (*p* < 0.05). Consistent with results of MTT assay, the capacity of colony formation with ERCC6L-knockdown HCC cells was significantly decreased than that of NC cells (Fig. 2d, *p* < 0.05).

The effects of silencing ERCC6L on HCC cell invasion were tested using a Transwell assay. As shown in Fig. 2e, the number of migratory shR-ERCC6L-transfected SMMC-7721 cells (64.67 ± 5.73) was significantly lower than the number of NC cells (157.67 ± 8.99, *p* < 0.05). Similar results were obtained for HuH-7 cells, with significantly fewer cells migrating through the Transwell membrane in the shERCC6L group (57.67 ± 9.10) than in the NC group (172.33 ± 6.13; *p* < 0.05). Collectively, these data indicated that downregulation of ERCC6L suppressed HCC cell proliferation and migration in vitro.

### Downregulation of ERCC6L induces cell cycle arrest and apoptosis in HCC cells

**Fig. 3:**
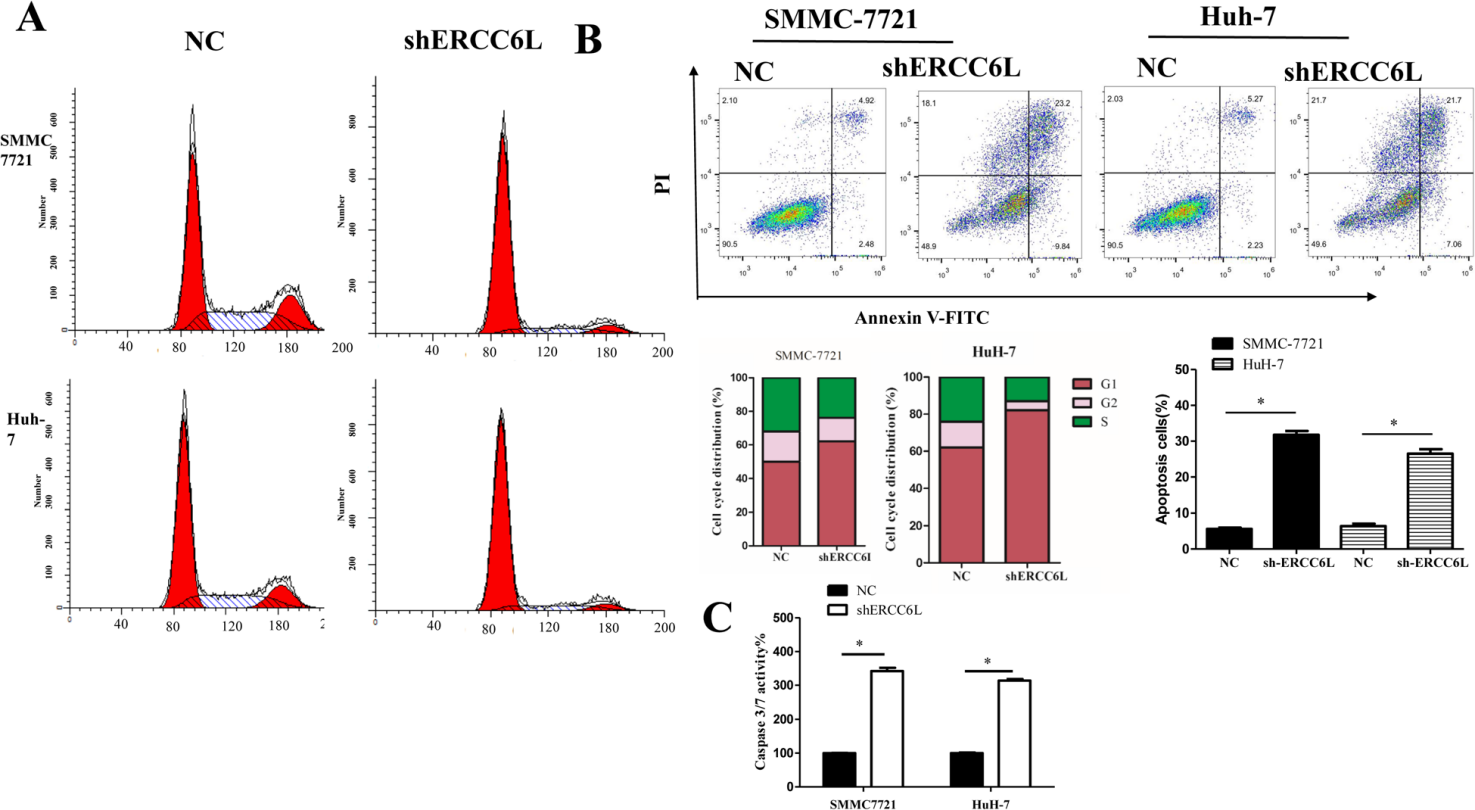
ERCC6L silencing induces cell cycle arrest and apoptosis in HCC cells. **a**. Cells were transfected with shERCC6L and NC for 24 h. Cell cycle progression was analyzed by PI staining with flow cytometry. The percentage of cells in each stage was quantified. Figures are representative of at least 3 independent experiments. Statistical differences were compared with NC, *p < 0.05. **b**. After transfected with shERCC6L and NC for 24 h, apoptosis cells were analyzed by Annexin V/FITC and PI staining with flow cytometry and apoptotic rates were quantified. Figures are representative of at least 3 independent experiments. **c**. Caspase 3/7 activation in SMMC7721 and HuH7 cells 24 h after transfection with shERCC6L or NC. Data are represent the mean ± SD of three independent experiments. *p < 0.05, **p < 0.001, indicates significant differences compared to the NC group

To determine the regulation of ERCC6L in cell cycle and apoptosis, we analyzed the cell cycle by flow cytometry. As shown in Fig. 3a, the percentage of cells in G1 phase was significantly higher in the shERCC6L-transfected SMMC7721 cells (81.73 ± 2.56%) than in the NC group (50.71 ± 3.21%; *p* < 0.05). Similarly, the percentage of cells in G0/G1 phase was significantly higher in shERCC6L-transfected HuH-7 cells (81.76 ± 3.72%) than in the NC group (62.24 ± 4.33%, *p* < 0.05). Thus it could be concluded that knockdown ERCC6L induces G1 phase cell cycle arrest.

Next, Annexin V-FITC and propidium iodide (PI) double staining was used to detect the apoptosis rate of SMMC7721 and HuH-7 cell by flow cytometry. As shown in Fig. 3b, the ratio of apoptotic SMMC7721 cells was dramatically higher in the shERCC6L-treated group (31.77 ± 1.090%) than in the NC group (5.597 ± 0.403%, *p* < 0.001) and the rate of HuH-7 cell apoptosis was also higher in the shERCC6L-treated group (26.53 ± 1.244%) than in the NC group (6.377 ± 0.680%, *p* < 0.001).

The apoptosis induction mechanism was analyzed by detecting caspases-3/7 activation with Caspase-Glo® 3/7 Assay Kit. The same tendency was observed as above, knockdown ERCC6L caused a significantly enhancing in caspase-3/7 activity than NC group (SMMC7721: 344.91 ± 0.64 vs. 100.0 ± 0.75, *p* < 0.001;Huh-7: 311.65 ± 1.58 vs.100.0 ± 3.47, *p* < 0.001) (Fig. 3c). These findings further demonstrated that knockdown of ERCC6L can effectively induce HCC cell apoptosis in a caspase-3/7-dependent manner and ERCC6L be involved in the regulation of the cell cycle and apoptosis.

### ERCC6L modulates HCC growth through PI3K/AKT and NF-κB signaling pathway

**Fig. 4:**
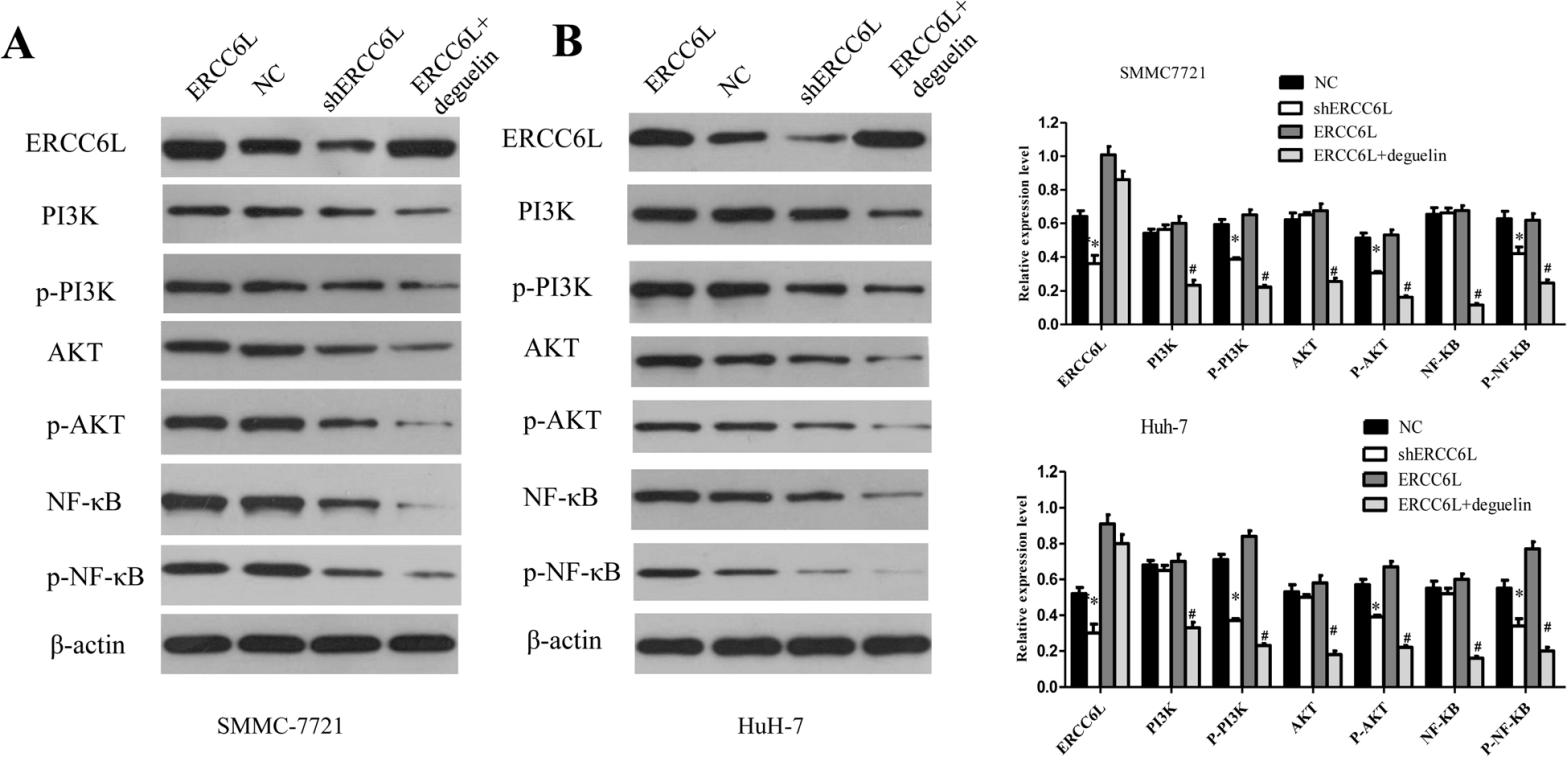
ERCC6L down-regulation inhibits PI3K/AKT and NF-κB pathway activation. SMMC7721(**a**) and HuH-7 cells (**b**) were transfected with shERCC6L, ERCC6L or NC for 72 h. Cell lysates were isolated and the expression of proteins involved in the PI3K/AKT and NF-κB pathway were analyzed by western blot. NC, cells transfected with empty vector; shERCC6L, cells transfected with shERCC6L; ERCC6L, cells transfected with ERCC6L; ERCC6L+ Deguelin, cells transfected with ERCC6L and treated with 5 μM/L deguelin for 4 h. *P < 0.05 vs. NC cells; ^#^*P* < 0.05 vs. ERCC6L. Dot plots represent one of three independent experiments. In the histogram, data are mean ± SD of three experiments

Previous studies showed that Phosphatidelinositol 3 kinase/Protein Kinase B (PI3K/AKT) signaling pathway is responsible for cancer proliferation, metastasis, angiogenesis, and survival [19–21]. NF-κB is a pleiotropic transcription regulating inflammation, cell survival and proliferation, which represents an important link between inflammation and tumorigenesis [22]. To explore whether PI3K/AKT and NF-κB involved in ERCC6L-induced HCC, we knockdown or overexpress ERCC6L expression in SMMC7721 and HuH-7 cells, respectively. Western blot analysis was performed to determine the expression of PI3K/AKT signaling-related molecules. As shown in Fig. 4a-b, compared with NC, shERCC6L decreased the levels of phosphorylated PI3K (p-PI3K), phosphorylated AKT (p-AKT) and phosphorylated NF-κB (p- NF-κB) (*p < 0.05), whilst the levels of non-phosphorylated PI3K, AKT and NF-κB remained unchanged. Furthermore, deguelin, a specific inhibitor of AKT was applied. ERCC6L- induced activation of PI3K/AKT signaling pathway were reversed by deguelin (5 μM/L, 4 h) (#*p* < 0.05). Deguelin (5 μM/L, 4 h) also blocked ERCC6L induced activation of NF-κB (Fig. 4a-b, Fig. S1). Altogether, regulation of ERCC6L confirmed that PI3K/AKT and NF-κB pathway were involved in the ERCC6L-mediated oncogenic function.

### ERCC6L down-regulation inhibits tumor growth in vivo

**Fig. 5:**
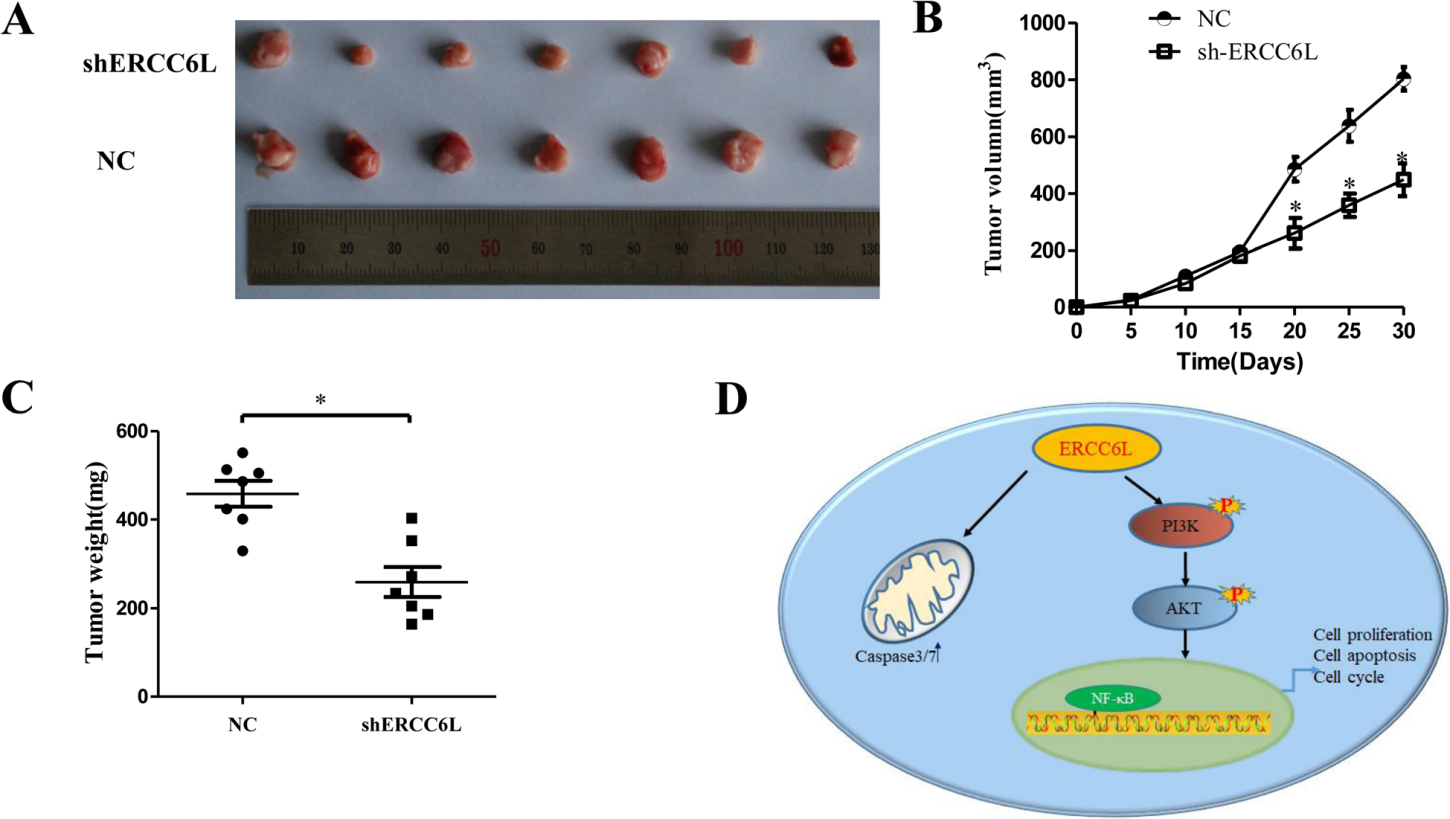
ERCC6L down-regulation inhibit tumor growth in vivo and hypothetical schematic diagram of mechanisms for HCC progression. (**a**) The mice was inoculated with either SMMC7721 cells stably expression shERCC6L or NC, and the dissected xenograft tumors from each group were photographed. (**b**) Tumor volume was measured every 5 days, and growth curves were created for each group. *p < 0.05 vs NC group. (c). Tumor weight at the endpoint (30 days). *p < 0.05 vs NC group. (d) The schematic diagram depicted in Fig. 5d has been created by the authors for this publication

To further determine the role of ERCC6L in tumor growth, we established xenograft tumor mouse model. Compared with that in NC group, tumor growth in the shERCC6L was significantly inhibited (*p* < 0.05, Fig. 5a-b). Moreover, the tumor weight in the shERCC6L group was significantly decreased than that in NC group (*p* < 0.05, Fig. 5c), showing that knockdown ERCC6L expression could retard HCC growth in vivo.

## Discussion

HCC is an aggressive malignancy, which metastasis, invasion, and recurrence are the main causes of death in HCC patients [23]. Rapid HCC progression and difficulty detecting early disease are major obstacles to curative treatment [24]. It is urgently required to screen novel diagnostic or prognostic biomarkers for targeted therapy. In this study, we found that higher ERCC6L expression levels are detected in tumor tissues than in adjacent normal tissues, suggesting that ERCC6L may play a crucial role in the progression of HCC. Moreover, high ERCC6L expression is a prognostic factor for reduced OS in HCC patients. Knockdown ERCC6L could inhibit HCC cell proliferation, invasion in vitro and vivo. In the aspect of molecular mechanism, we confirmed that PI3K/AKT and NF-κB pathway were involved in the regulation of ERCC6L. Taken together, our findings reveal that ERCC6L overexpression correlates with the development of the malignant tumor and may be an effectively prognostic factor and potential therapeutic target for HCC patients.

Increasing evidences have demonstrated that ERCC6L is an important oncogene in tumor progression. Pu et al. [10] observed that knockdown ERCC6L expression inhibited the proliferation of breast and kidney cancer cell in vitro *and vivo*. Upregulated ERCC6L mRNA was notably correlated with the progress of tumor and associated with poorer outcomes in breast and kidney cancer patients. They also found that RAB31 may be as ERCC6L downstream protein involved in the progression of cancer via phosphorylated MAPK and CDK. Zhang et al. [25] analyzed a tissue microarray containing 150 renal cell carcinoma (RCC) samples showed that compared with adjacent tissue, ERCC6L was overexpressed in the tumor tissues, and abnormal expression was positively correlated with the cancers progression. Meanwhile, knockdown ERCC6L expression inhibited RCC cells viability and induced apoptosis accordingly. In terms of mechanism, they confirmed that MAPK signaling pathway was involved in the regulation of ERCC6L on cellular process of RCC. Zhong et al. [11] showed that four genes (ERCC6L, AHCY, STK33, and NCAN) have been identified to increase in the neuroblastoma and predicted poor prognosis of neuroblastoma patients. Of these, ERCC6L was an independent prognostic factor of overall survival and event-free survival. Furthermore, they identified that some special genes such as MAD2L, CCNB1 and BIRC5, which had a close relationship with ERCC6L were involved in the cell cycle pathway, thus ERCC6L may play an important role in neuroblastoma. Recently, Xie et al. [26] found that ERCC6L was abnormal overexpressed in colorectal cancer (CRC) tissues and cell lines, and reducing ERCC6L expression in CRC cells significantly inhibited the proliferation, cell cycle progression, and arrested cell cycle at G0/G1 phase. These findings demonstrated that ERCC6L may exert an essential role in tumor growth and may be an efficient target for tumor detection, diagnosis and therapy.

In the current study, we evaluated the expression of ERCC6L in HCC patients and demonstrated that ERCC6L levels were overexpressed in the tumor tissues than in paired tumor-adjacent tissues. Meanwhile, ERCC6L expression levels were positively associated with clinicopathological characteristics, including Edmondson stage, tumor encapsulation, and female gender. Furthermore, abnormal expression of ERCC6L was associated with shorter OS compared with low ERCC6L expression in patients, indicating that ERCC6L expression is markedly significant for the prognosis of HCC patients. Epidemiological studies of HCC have showed that it was notably more prevalent in male than in female, with a male-to- female ratio ranging from 2:1 to 8:1 [27]. We also found that ERCC6L expression was higher in man than that of women. Although in line with this trend, however the proportion of female patients in our sample was lower, than it may need to further expand the sample size for further research. High ERCC6L expression was also found in the patients with non-tumor encapsulation or III-IV pathological stage, which often indicating highly invasive, metastatic, and poor prognosis.

Similarly, the level of ERCC6L mRNA and proteins expression was also elevated in HCC cell lines than in normal human liver cells. Then, we revealed that interfering with ERCC6L expression with shRNA in SMMC-7721 and HuH-7 cells significantly inhibited their proliferation in vitro and in vivo. Apoptosis is a cellular mechanism characterized with programmed cell death and is result of chain of reaction modulated by many effectors for regulating coordinates cell proliferation and cell death [28]. Previous studies have observed that caspase3 is considered as a crucial executioner of apoptosis and activated caspase3/7 may cleave the majority of polypeptides which undergo proteolysis in cells, and was an independent prognostic factor for tumor [29] . Knockdown ERCC6L promoted the G1 phase cell arrest and increased the proportion of apoptosis cells by caspase3/7 dependent manner. Collectively, these data indicated that ERCC6L may function as an oncogene in the occurrence and development of the HCC.

Numerous studies have shown that the PI3K/AKT signaling pathway plays a crucial role in malignant transformation and the subsequent growth, proliferation, and metastasis of human tumors [30, 31]. The downstream effectors of abnormal activated PI3K/AKT pathway may contribute to its role in progression of tumor growth, apoptosis, altered endothelial cell function, and angiogenesis, such as NF-κB and mTOR [32–35]. In a clinical study, PI3K/AKT pathway activation was associated with tumor progression and the reduced survival of HCC patients [36]. Caspases-7 and caspases-3 are involved in the initiation and execution of apoptosis, respectively. Activated AKT can phosphorylate caspases-7 and caspases-3 to prevent caspase-9 and caspase-3 activation [37]. We investigated the mechanism by which ERCC6L shRNA inhibited HCC progress. Results revealed that knockdown-ERCC6L expression reduced the level of phosphodiesters related to the PI3K/AKT and NF-κB. Furthermore, the rescue experiments clarified that AKT inhibitor attenuated the enhancement of upregulating mediated p-PI3K/p-AKT and p-NF-κB protein expression. These data suggested that ERCC6L may regulate HCC via PI3K/AKT pathway (Fig. 5d). This may provide a new direction for studying ERCC6L function.

## Conclusions

This study demonstrated that the ERCC6L expression was upregulated in HCC tumor tissue, which exhibited closely associated with tumor progression and poor prognosis. Furthermore, aberrant expression of ERCC6L promoted tumorigenesis via the PI3K/AKT and NF-κB pathway. Hence, ERCC6L may be a potential therapeutic target for HCC.

## Supplementary information

**Additional file 1: Figure S1.** Uncropped full-length blot images for Fig. 2b, 4a, b. The cropped blots were marked with red frame.

## Data Availability

All datasets generated for this study are included in the manuscript and/or the supplementary files.

## Abbreviations

ERCC6L: Excision Repair Cross-Complementation group 6-like
HCC: Hepatocellular Carcinoma
PI3K: Phosphoinositide 3-kinase
AKT: Protein kinase B
NF-κB: Nuclear factor-kappa B
OS: Overall survival rates
