# Supplementary material for: ERCC6L promotes the progression of hepatocellular carcinoma through activating PI3K/AKT and NF-κB signaling pathway
Source: BMC Cancer. 2020 Sep 5;20:853. doi: 10.1186/s12885-020-07367-2 (PMC7487553; doi:10.1186/s12885-020-07367-2)

Original Blots Figure 2B

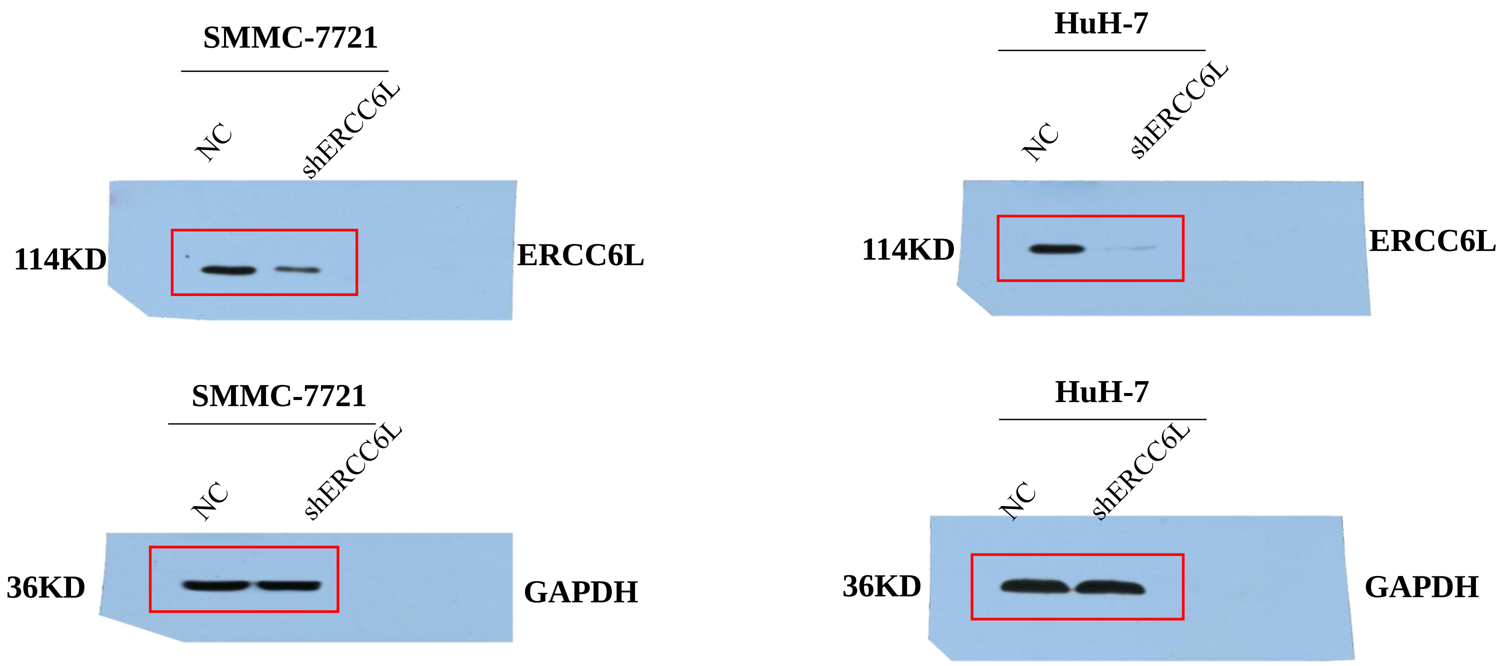

Original Blots Figure 4A

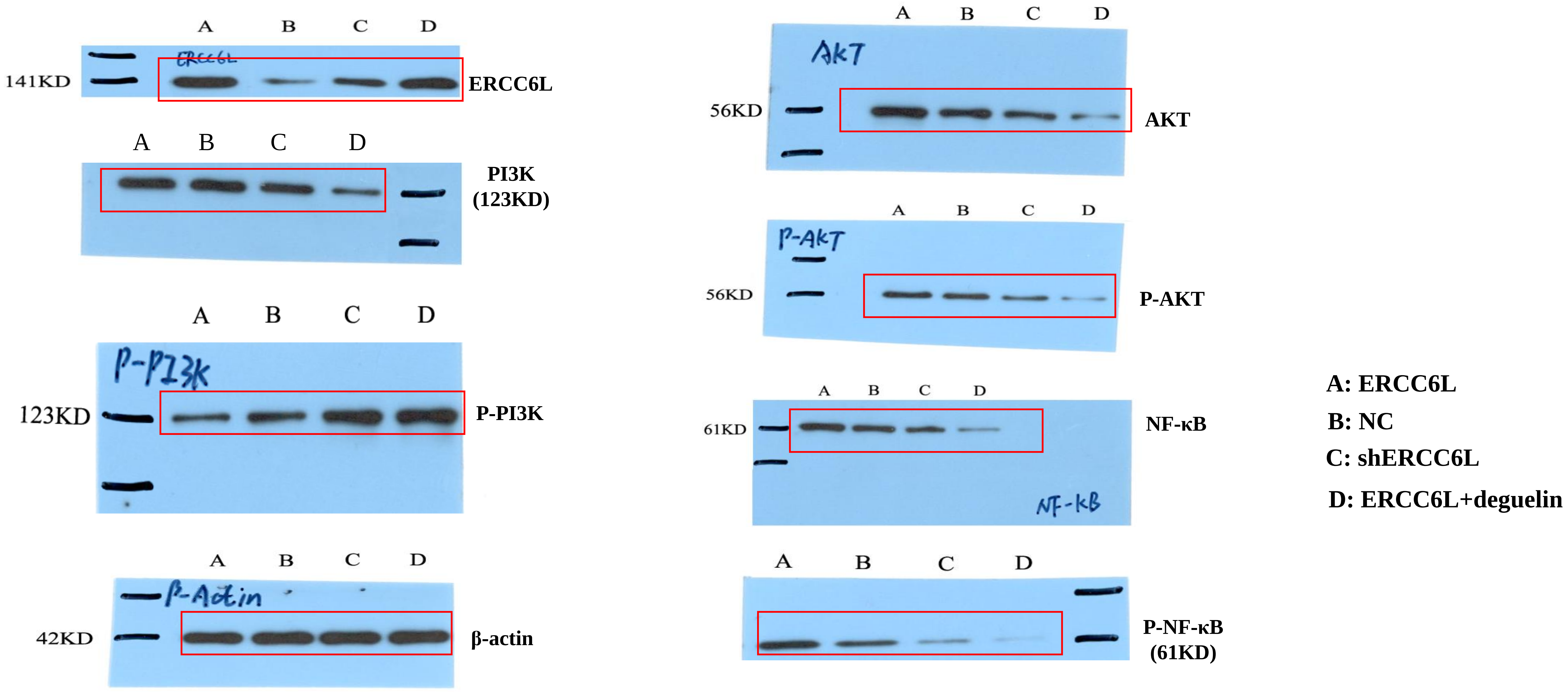

Original Blots Figure 4B

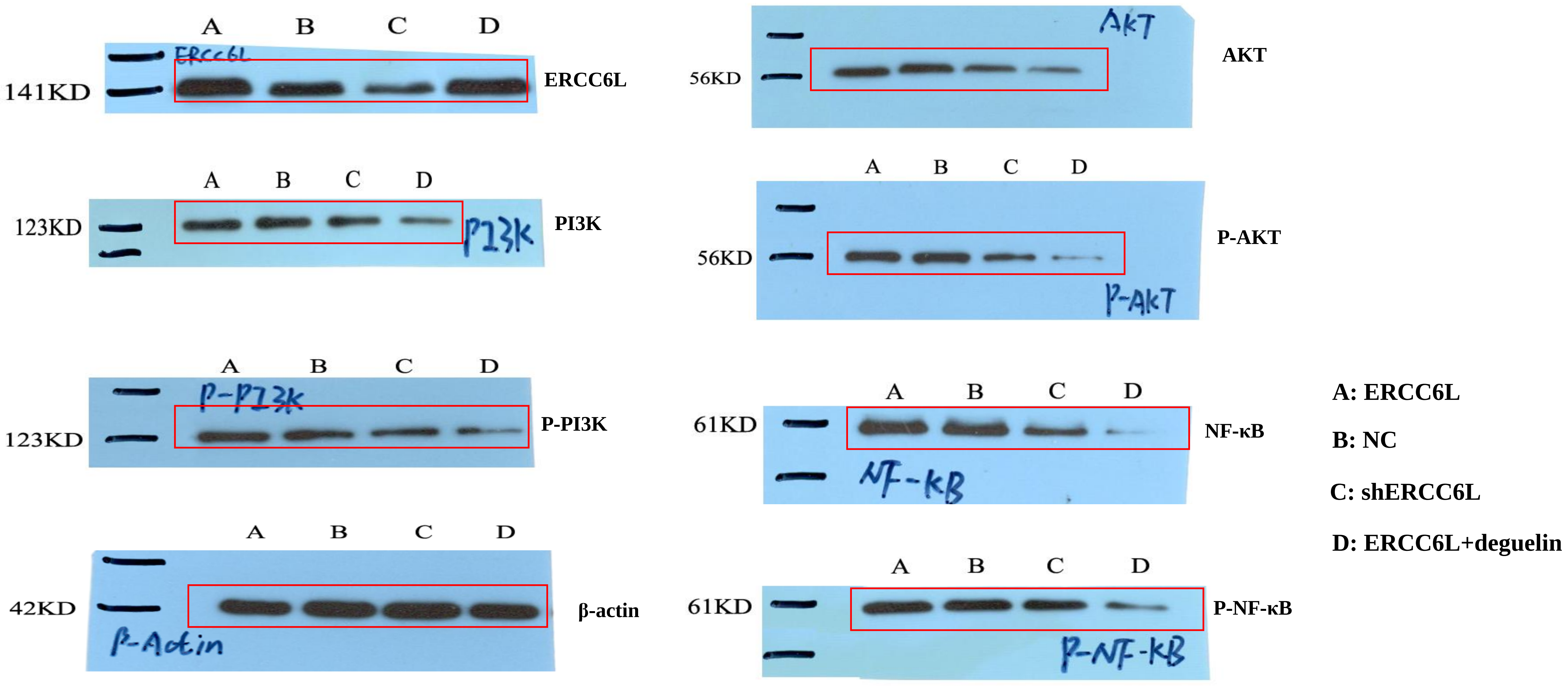

Supplement: Supplementary file 1 — Additional file 1: Figure S1. Uncropped full-length blot images for Fig. 2b, 4a, b. The cropped blots were marked with red frame. [file 12885_2020_7367_MOESM1_ESM.pdf]
